# A rare instance of latent systematic error in volumetric-modulated arc therapy with field-extended multi-isocentre irradiation leading to a serious dose-delivery accident

**DOI:** 10.1093/bjrcr/uaae021

**Published:** 2024-07-17

**Authors:** Takashi Hanada, Junichi Fukada, Yutaka Shiraishi, Kayo Yoshida, Naoya Sakanoue, Kohei Oguma, Toshio Ohashi, Naoyuki Shigematsu

**Affiliations:** Department of Radiology, Keio University School of Medicine, Tokyo 160-8582, Japan; Department of Radiology, Keio University School of Medicine, Tokyo 160-8582, Japan; Department of Radiology, Keio University School of Medicine, Tokyo 160-8582, Japan; Department of Radiology, Keio University School of Medicine, Tokyo 160-8582, Japan; Department of Radiology, Keio University School of Medicine, Tokyo 160-8582, Japan; Cancer Center (Radiotherapy Unit), Keio University School of Medicine, Tokyo 160-8582, Japan; Department of Radiation Oncology, Tokyo Saiseikai Central Hospital, Tokyo 180-0073, Japan; Department of Radiology, Keio University School of Medicine, Tokyo 160-8582, Japan

**Keywords:** radiotherapy, volumetric-modulated arc therapy, multi-isocentre, dose junction, systematic error, fluence map

## Abstract

Volumetric-modulated arc therapy (VMAT) with field-extended multi-isocentre irradiation (VMAT-FEMII) is an effective irradiation technique, particularly for large planning target volumes in the craniocaudal direction. A variety of treatment planning techniques have been reported to reduce the dosimetric impact. However, there is no guarantee that unexpected latent systematic errors would not occur. Herein, we report the experience with a rare case that could have led to a serious VMAT-FEMII-related accident. A patient with uterine cervical carcinoma was scheduled for VMAT-FEMII to the whole pelvis and the para-aortic lymph node region. A combination of the two sets of field groups with different isocentres was planned: one to cover the para-aortic lymph nodes and the other to cover the whole pelvis. Measurements based on the pretreatment dose delivery quality assurance (QA) revealed an unexpected overdose of >20% in the field overlap region. This overdose phenomenon is not reflected in the calculated dose distribution in the radiotherapy treatment planning system. Therefore, the plan was altered; a homogeneous dose distribution inside the dose junction was achieved. Several analyses were performed to elucidate the overdosing phenomenon. However, no conclusive answer was found to why non-reflection at the calculated dose distribution was found. The limitations to VMAT-FEMII are primarily related to systematic errors in the positional setup from patient-derived and/or mechanical sources. However, this report highlights a rare case of overdosing caused by inverse optimization and dose calculation. We recommend checking the aperture status of the jaw and multi-leaf collimator at each control point of the treatment plan and using a high-resolution image measurement system on a VMAT-FEMII QA to confirm the dose junction status.

## Background

Volumetric-modulated arc therapy (VMAT) was initially proposed as an irradiation technique in 1995 for external beam radiotherapy.^1^ This technique involves treating patient with one or more gantry arcs with a continuously varying beam aperture, gantry speed, and dose rate.^2^ In addition, technical approaches for VMAT promise to maximize the benefit of intensity-modulated delivery dose by treating patient with the widest range of beam orientations in the shortest possible time by solving a large-scale nonconvex optimization problem describing the ideal beam delivery fluence map.

The widest range of the maximum field size per isocentre is limited owing to the mechanical operation range of the collimators (referred to as jaws) and multi-leaf collimators (MLC). To implement an extended treatment field that overcomes machine limitations, the irradiation area requires an overlapping combination of multiple fields with different multiple isocentres. This technique known as VMAT with field-extended multi-isocentre irradiation (VMAT-FEMII) is effective, particularly in the treatment of large dimensions of the planning target volume in the craniocaudal direction (CCD).^3–8^

Regarding VMAT-FEMII, field overlapping generates dose junctions; this leads to specific issues relating to position accuracy and systematic errors of under- or over-dosing phenomena.^9^^,^^10^ Most of these systematic errors tend to arise from positional setups, including patient-derived and/or mechanical processes. Therefore, a variety of treatment planning techniques have been reported to reduce the dosimetric impact.^11–15^ However, there is no guarantee that other latent systematic errors will occur. Herein, we report our experience with rare cases that could lead to serious treatment accidents related to dose delivery.

## Case presentation

**Figure 1.**
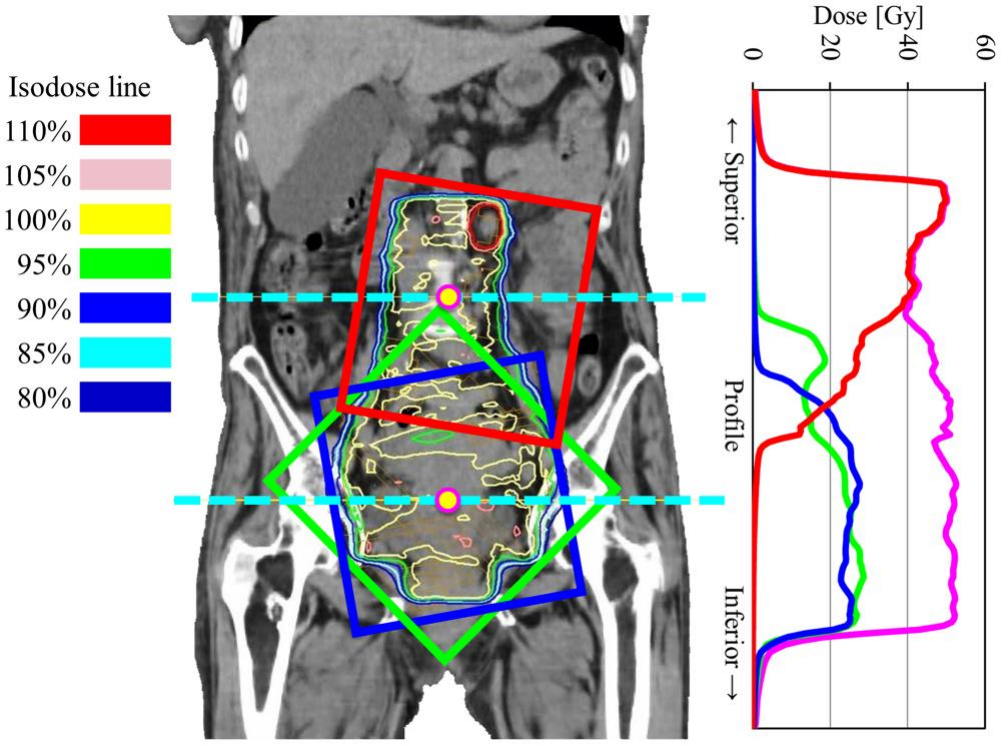
Coronal view beam arrangement for each fieldset planned with RTPS. Isodose line of 100% equals to 50.4 Gy. Light blue dash lines represent the line of the isocentre plane. Field^PAN^ (red rectangle) covers the para-aortic lymph nodes region and Field^WP^ (green and blue rectangle) covers the whole pelvis region. The profiles represent the dose that passes through the isocentre in CCD. The colour line in the profile corresponds to each field colour (magenta describes the total dose). Abbreviations: RTPS: radiotherapy treatment planning system; CCD: craniocaudal direction.

In this study, we present a case involving irradiation of the whole pelvis plus para-aortic lymph nodes (PANs) region for uterine cervical carcinoma treated with VMAT-FEMII on a TrueBeam (Varian Medical Systems, Palo Alto, CA, United States) linear accelerator (LINAC). The treatment plan was created using Eclipse ver. 16.10 (Varian Medical Systems, Palo Alto, CA, United States) radiotherapy treatment planning system (RTPS) using a 10 MV photon beam. The irradiation technique of VMAT-FEMII was applied to conduct simultaneous integrated boost irradiation, achieving 50.4 Gy in the whole pelvis and PANs and 60 Gy in swollen lymph nodes in 28 fractions. A combination of two sets of field groups in different isocentres was configured, one to cover the PANs region (Field^PAN^) and the other to cover the whole pelvic region (Field^WP^). Figure 1 illustrates the configuration of the irradiation fields and Table 1 details the treatment planning, including field information. The plan field of Field^PAN^ and Field^WP^ was optimized simultaneously using a photon optimizer algorithm. Furthermore, the associated dose distribution was computed using the AcurosXB dose calculation algorithm with a 2-mm dose-grid resolution. In addition, the overlap technique of the automatic feathering algorithm, which creates a homogeneous dose distribution inside the dose junction, was optimized to address the problems of positional setup uncertainties in treatments with VMAT-FEMII.^15^

**Table 1.** Data on the treatment plan.

	Field^PAN^	Field^WP^	
Number of consistent fields	1	2	
LINAC	Varian TrueBeam		
RTPS	Eclipse version 16.10		
Energy (MV)	10		
Dose rate (MU/min)	600		
Jaw tracking	ON		
Field size (X1/X2/Y1/Y2) (cm)	7.7/5.4/7.6/6.5	6.9/8.4/6.8/7.4	8.2/7.9/6.8/7.4
Dose calculation algorithm	AcurosXB		
Dose grid resolution (mm)	2		
Gantry rotation (degrees)	181.0-179.0	181.0-179.0	179.0-181.0
Collimator rotation (degrees)	10	45	350
Patent support angle (degrees)	0	0	0
Monitor unit (MU)	430	168	129

Abbreviations: RTPS = radiotherapy planning system; LINAC = linear accelerator; MU = monitor unit.

**Figure 2.**
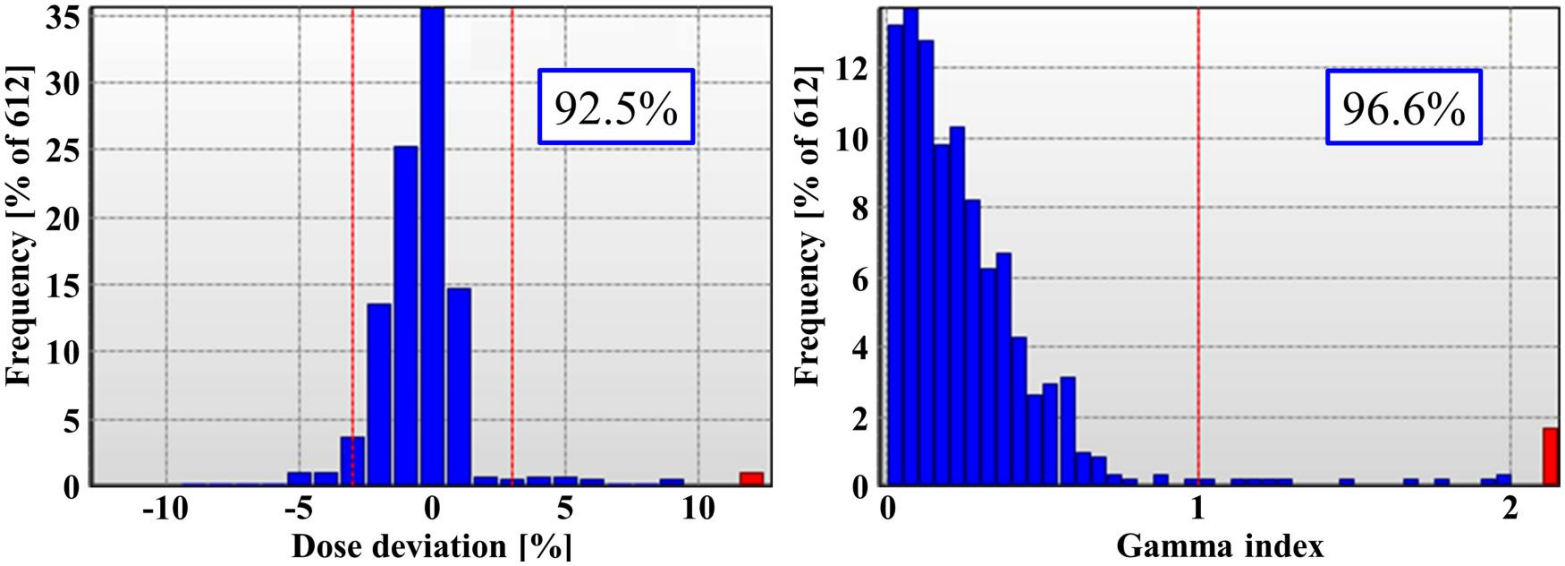
The result of dose difference (right) and GIPR (left) on FieldP^AN^ measured by Delta4 diode system. Percentage values in the blue box show the pass ratio of each criterion (red lines). Several points exceeding 10% dose differences and gamma index 2 are observed (red bars). Abbreviation: GIPR = gamma index pass ratio.

**Figure 3.**
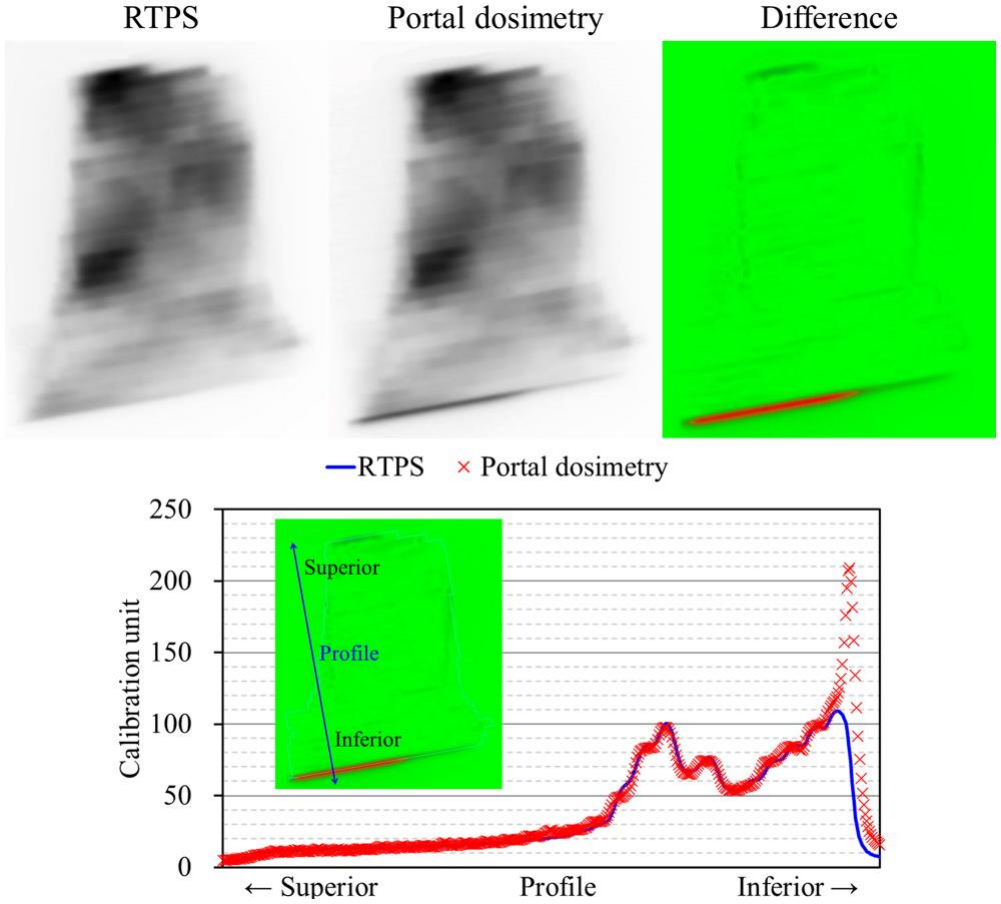
The results of difference in Field^PAN^ from calculated and measured fluence map. The red colour area in the fluence map describes the large discrepancies. The profiles are also shown. Abbreviation: RTPS = radiotherapy treatment planning system.

**Figure 4.**
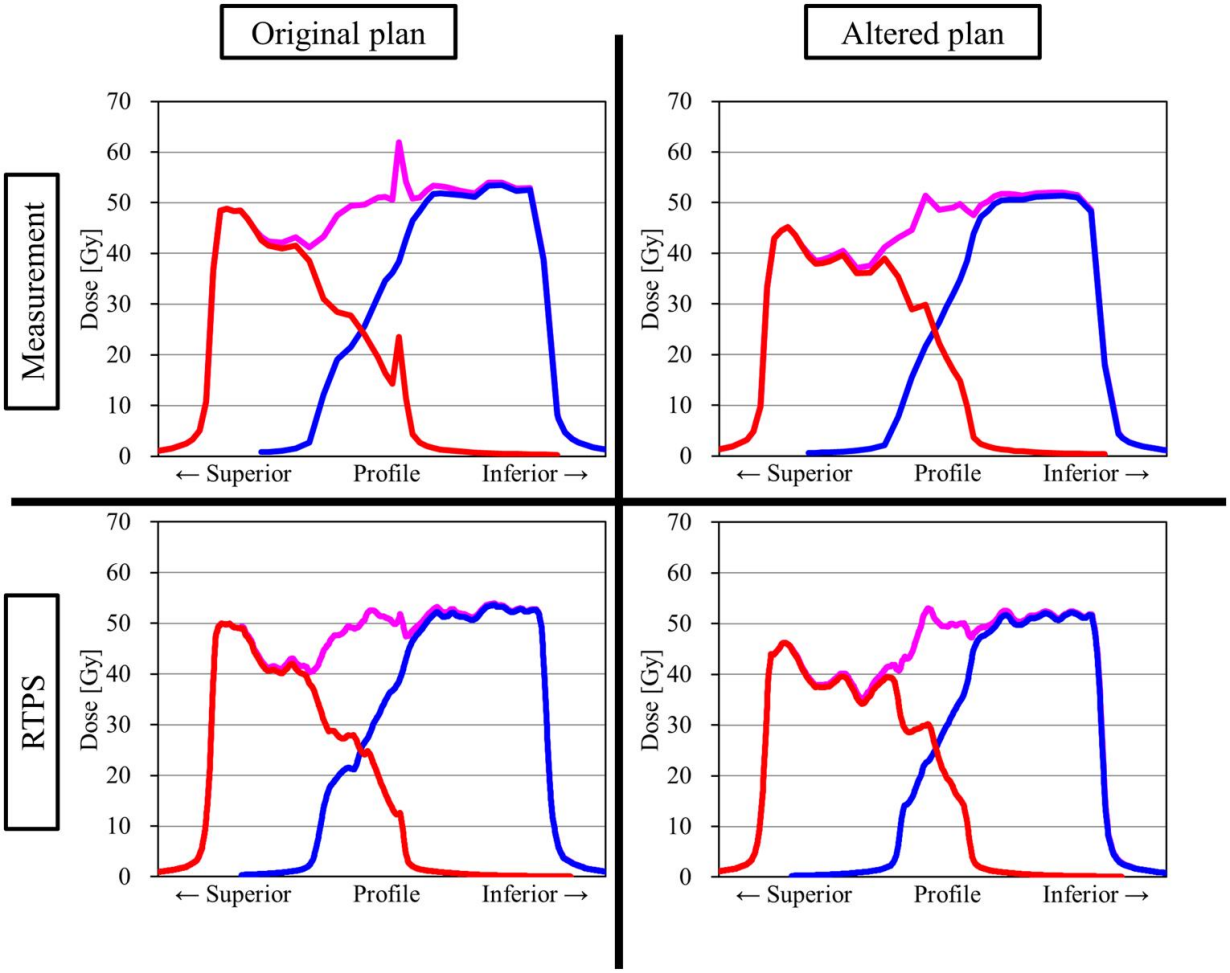
The dose profile passing through the isocentre in CCD is obtained from the Delta4 diode system. The over-dosing phenomenon is suppressed in a new plan and homogeneous dose distribution inside the dose junction is achieved. The red and blue lines represent the dose profile of Field^PAN^ and Field^WP^, respectively (magenta describes the total dose). Abbreviation: CCD = cranio-caudal direction.

Measurements of the pretreatment dose delivery quality assurance (QA) were performed using a Delta4 (ScandiDos AB, Uppsala, Sweden) diode array system.^16^ The analyses were performed using the global gamma index pass rate (GIPR) with 3%/2 mm (20% dose threshold), which has already been established in our clinic for all VMAT QA. The gamma index was computed by the percentage dose difference and distance-to-agreement (DTA) at each detector point for a given set of doses and DTA criteria. The ratio of passing points to all points was calculated as the GIPR. In this case, the GIPR results of Field^PAN^ and Field^WP^ were 96.6% and 100.0%, respectively. However, several points exceeding 10% of the dose differences and 2 of the gamma index were observed from the results of Field^PAN^ (Figure 2). Additional measurements were performed to obtain the entire dose delivery information using an electronic portal imaging device (EPID) via the portal dosimetry (PD) QA procedure for comparison with beam delivery fluence maps obtained from the RTPS. The PD measurements revealed that the points of large discrepancies were generated at the field edge, which was the relevant region of the dose junction with Field^WP^ (Figure 3). If this plan was to be adopted for actual treatment, the delivery dose in the overlapping region with Field^WP^ would receive an unexpected overdose of more than 20%. Therefore, we decided to replan; a homogeneous dose distribution inside the dose junction was achieved by the altered plan (Figure 4).

## Discussion and conclusions

**Figure 5.**
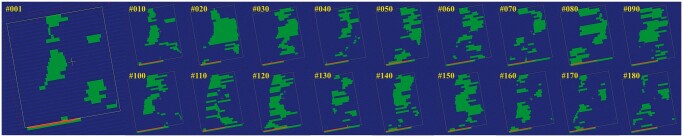
The aperture status of jaw and MLC on Field^PAN^ at each control point. The positions of the jaw (yellow line) and MLC (blue area) were mapped on a fluence map (Difference) in Figure 3. The MLCs in the over-dosing area were constantly opened. The numbers are the indices of control points. Abbreviation: MLC = multi-leaf collimator.

**Figure 6.**
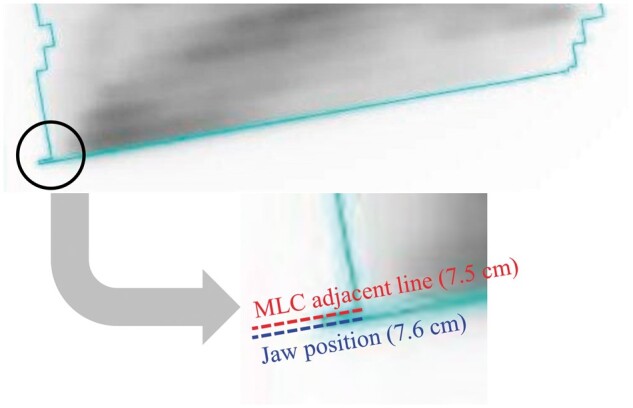
Illustration of field edge generated by the region of over-dosing phenomenon in Field^PAN^. The positions of MLC (light blue line) are mapped on the fluence map (RTPS) in Figure 3. Abbreviations: MLC = multi-leaf collimator; RTPS = radiotherapy treatment planning system.

**Figure 7.**
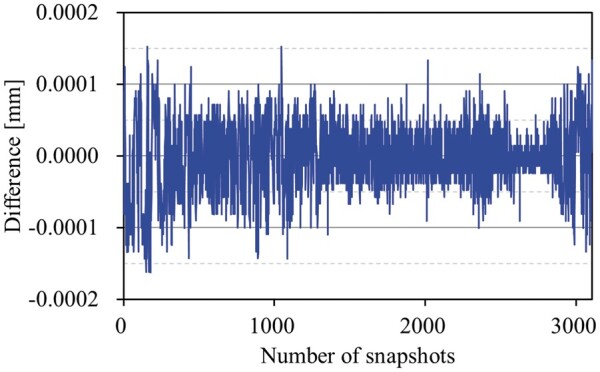
The result of the difference in Y1 jaw positions between RTPS and actual beam delivery from a trajectory log file. Abbreviation: RTPS = radiotherapy treatment planning system.

The main issue in this case was that the overdosing phenomenon was not accurately calculated in the dose distribution at the RTPS. This was elucidated in details by checking the aperture status of the jaw and MLC and analysing the positions at each control point on Field^PAN^, revealing that the MLC was constantly opened in the overdosing area (Figure 5). Moreover, this overdosing area was generated in a narrowly spaced area (1.0 mm) between the Y1 jaw position and adjacent MLC (Figure 6). We suspected maladjustment of the Y1 jaw positions. However, no problem of quality in jaw positions was confirmed by ionization chamber measurements with a water tank and an EPID via PD. Next, we focused on trajectory log files, which record information during the delivery of a beam in a binary manner, that is, the expected and actual status of the TrueBeam beam delivery parameters.^17^ We analysed the Y1 jaw positions from the trajectory log files. However, no problems were detected (Figure 7). Focusing on the MLC position errors, the picket fence test, which is used for routine MLC QA, was performed, resulting in detecting no problem of quality. Furthermore, we considered that the error of MLC position would not be the direct cause of overdosing since the overdose phenomena had appeared along with directions of MLC movements. Using another approach, changing the dose grid resolution and dose calculation algorithm in RTPS settings does not account for the overdosing phenomena.

**Figure 8.**
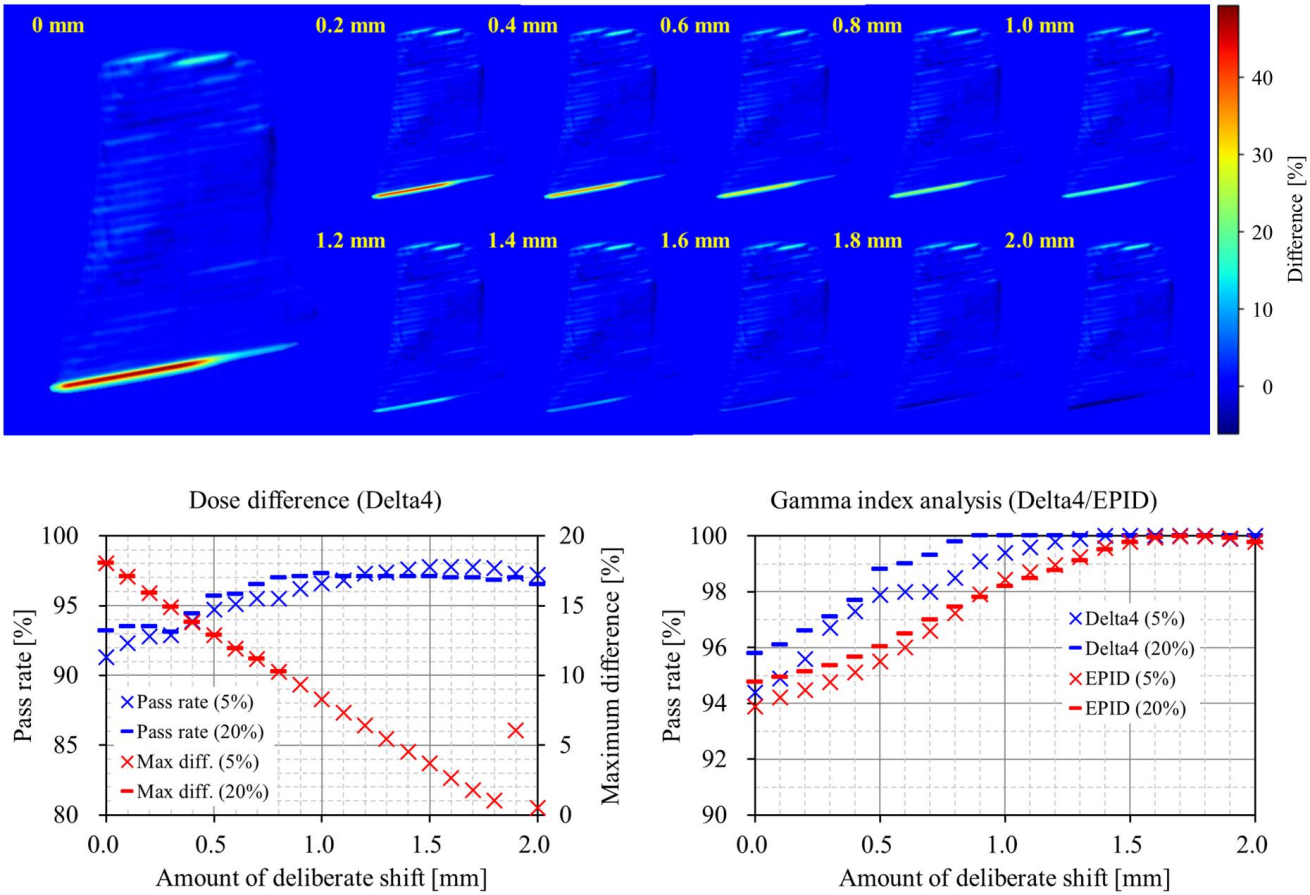
The results of the difference of the fluence map (top), DDPR (bottom-left), and GIPR (bottom-right) from the Field^PAN^ pseudo plan measurements using the Delta4 diode system (bottom-left and bottom-right) and EPID (top and bottom-right). The values on the fluence maps are the amount of deliberate shift of Y1 jaw positions from the original plan value. The bracket values on the graphs are the dose thresholds. Abbreviations: DDPR = dose difference pass rate; GIPR = gamma index pass rate; EPID = electronic portal imaging device; Max = maximum; diff. = difference.

Performing further analysis to elucidate the over-dosing phenomena, we estimated the sensitivity of the dose difference pass rate (DDPR) and GIPR that were affected by the systematic error of jaw position. The Field^PAN^ plan was copied and several pseudo-plans were created using the developed in-house C++ program code. These pseudo-plans deliberately simulated the systematic errors of the Y1 jaw positions in 0.1 mm incrementations. The measurements of the pseudo-plans were performed field-by-field using the Delta4 diode system and EPID (Figure 8). Based on these measurements, a pseudo-plan of a 1.0-1.5 mm inner shift in the Y1 jaw positions met the goal of passing the 3%/2 mm VMAT QA criteria. However, the Y1 jaw positions were not miscalibrated as they were appropriately confirmed from the chamber measurements with a water tank and EPID via PD.

**Figure 9.**
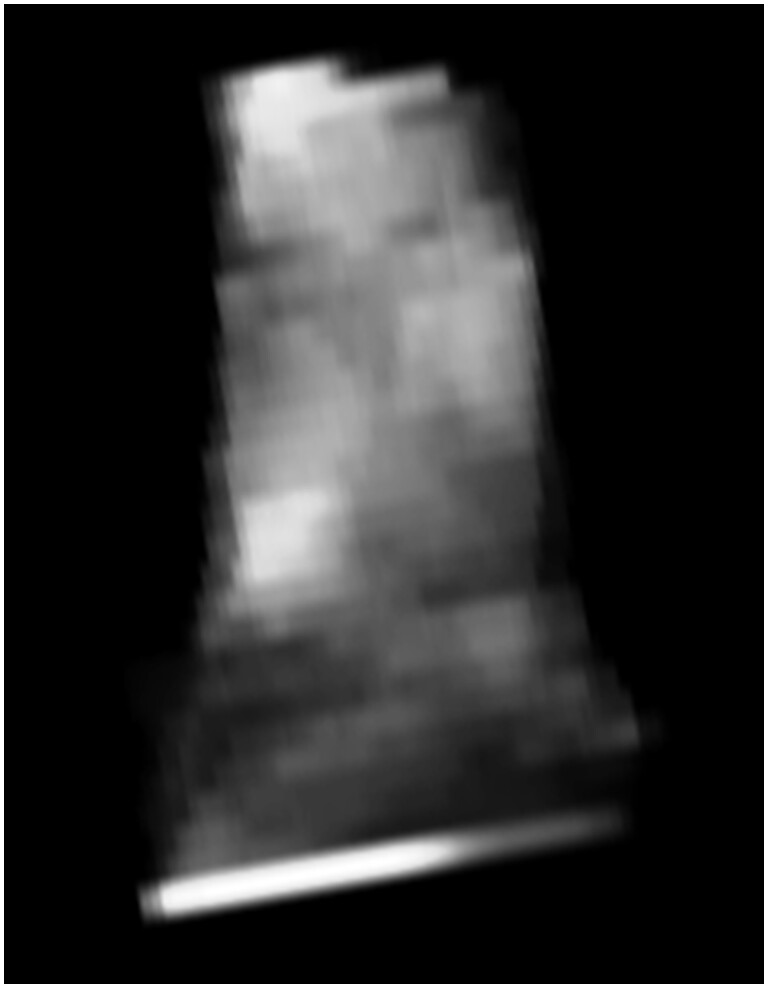
The planned-expected fluence map is constructed from the trajectory log file representing the expected MU delivery information. The white-coloured area in the fluence map describes the large MU delivery. Abbreviation: MU = monitor unit.

Another approach reveals interesting findings. The planned expected beam delivery fluence map was constructed with the trajectory log file by image calculation, which overlaid the fluence map consisting of a monitor unit (MU) and the MLC positions at all control points (Figure 9). Using the constructed planned-expected beam delivery fluence map, which represents the designed delivery dose (MU) information, a large fluence of the delivery dose associated with overdosing phenomena was demonstrated in the plan information from the MU and MLC positions.

**Figure 10.**
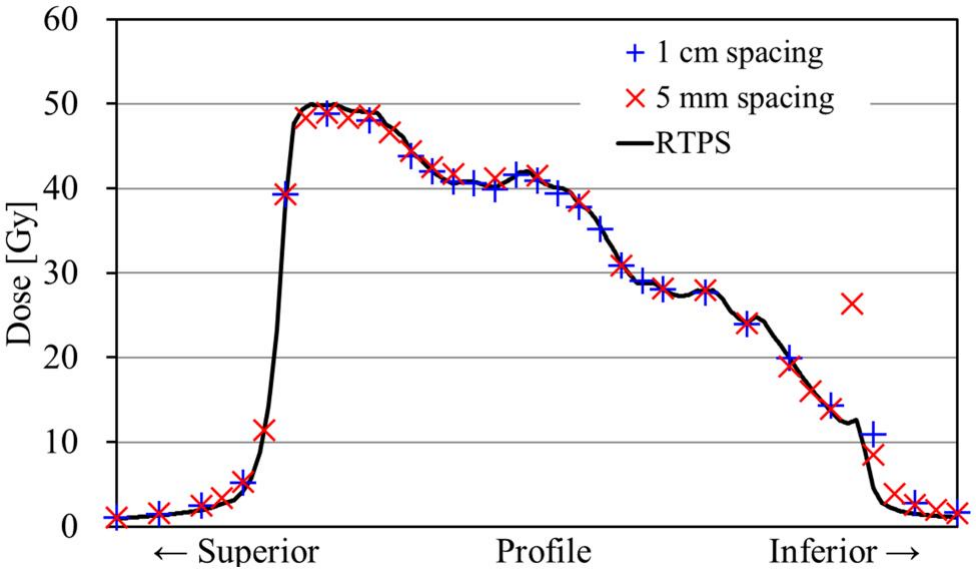
Dose profile passing through the isocentre in CCD from the measurements with 1 cm and 5 mm detector spacing using the Delta4 diode system. The over-dosing phenomena were not detected with the 1 cm detector spacing measurements. Abbreviation: CCD = cranio-caudal direction.

In practice, the calculated dose distribution created by the RTPS does not reflect the overdosing phenomenon. Actual measurements have confirmed this phenomenon. Of note, using a multidimensional detector for verification, there is a possibility that the event of a dose difference occurring at the edge of the irradiation field would be buried and the event would not be detected. Moreover, the potential exists for similar errors to occur in other single-isocentre VMAT plans. These were dependent on the dose threshold of the detected signal and positions of the discretely placed detectors to be measured (Figures 8 and 10). Regarding typical single-isocentre VMAT plans, minor field edge discrepancies might not be a critical problem because they affect the outer PTV contour. However, VMAT-FEMII with overlapping fields could deliver overdoses to the inner PTV region, leading to serious consequences. Therefore, high-resolution image measurement systems such as film and EPID are suitable for confirming the dose junction status and should be added to VMAT-FEMII QA to prevent such issues. In this case, we did not analyze the films in this study, opting for using EPID with portal dosimetry to assess the overlap region. Although film offers slightly higher resolution (0.17 mm in 150 dpi scanning), EPID’s resolution (0.34 mm) was sufficient for our purposes.

During the initial occurrence of the issue of overdosing phenomenon, we received brief instructions from the vendor regarding its validation methods, such as changing the dose calculation grid size. Furthermore, we performed the additional analysis meticulously, conducting thorough verifications and reported the results to the vendor. Despite these efforts, the root cause of the problem remained elusive. Consequently, it was determined that the issue would be revisited if it arose again. However, there is a current situation of the issue has not reappeared to date.

Due to its advantages of shorter treatment times and potentially reduced motion uncertainty, VMAT-FEMII has been commonly applied in clinical practice. Inversely optimized VMAT planning could achieve the intended gradient and smooth dose junction with a simple approach and allow for the concurrent optimization of multiple overlapping fields without explicit control of the junction dose, relying on the optimization algorithm. However, as an inversely optimized technique, the meticulous planning of field junctions complicates the VMAT planning process and can lead to unexpected latent systematic errors. The limitations of VMAT-FEMII are primarily reported on systematic errors of the positional setup related to patient-derived and/or mechanical errors. However, our report highlighted the rare possibility of dose delivery accidents caused by inverse optimization and dose calculation processes. Consequently, regarding irradiation using VMAT-FEMII, we recommend checking the aperture status of the jaw and MLC at each control point in the treatment plan. Furthermore, VMAT-FEMII QA, which uses a high-resolution image measurement system, should be used to confirm the dose junction status.
